# Effect of emergency obstetric care and proximity to comprehensive facilities on facility-based delivery in Malawi and Haiti

**DOI:** 10.1371/journal.pgph.0000184

**Published:** 2022-02-02

**Authors:** Chioma T. Amadi-Mgbenka, Luisa N. Borrell, Heidi E. Jones, Andrew Maroko, Francisco Bolumar

**Affiliations:** 1 Department of Epidemiology & Biostatistics, CUNY Graduate School of Public Health and Health Policy, New York City, New York, United States of America; 2 Universidad de Alcalá, Madrid, Spain; 3 Department of Environmental and Occupational Health Sciences, CUNY Graduate School of Public Health and Health Policy, New York City, New York, United States of America; Southern Cross University, AUSTRALIA

## Abstract

Proximity of households to comprehensive obstetric care is a key determinant for preventing maternal mortality due to obstetric emergencies. The relationship between proximity to comprehensive care and facility delivery is further complicated by the use of varied methods in measuring facility obstetric capacity–which may misrepresent the real scenario of obstetric care availability in a service environment. We investigated the joint effects of proximity and two emergency obstetric care assessment (EmOC) methods on women’s place of delivery in Malawi and Haiti. Household level and health facility data were obtained from the 2013–2018 Demographic and Health Surveys and Service Provision Assessment surveys. Records of women aged 15 to 49 years who had a childbirth in the last 5 years were linked to obstetric facilities within 5km, 10km and 15km from their households using Kernel Density Estimation. Log-binomial models were fitted to estimate the joint effects of proximity to comprehensive facilities on place of delivery and two EmOC methods (1. the facility’s recent performance of signal functions only, and 2. a composite index of obstetric care), and whether this varied by urban/rural setting. Proximity to comprehensive facilities was significantly associated with facility delivery in Malawi among women living 5km of a comprehensive facility (using EmOC method 2), in addition, living further (15km) from facilities with high capacity of EmOC was associated with reduced likelihood for facility delivery in urban settings in stratified analyses. In contrast, positive associations were present in Haiti in both urban and rural settings, with the likelihood of facility delivery being higher with greater proximity of women to comprehensive facilities, regardless of methods to define EmOC. Women living within 5km of a comprehensive facility in Haiti were the most likely to deliver in facilities based on EmOC method 1 (APR: 1.81, 95% CI 1.56, 2.09). Findings from Malawi elucidates the relevance of context and suggests the need for research in diverse settings.

## Introduction

Despite global efforts to mitigate maternal mortality, more than 300,000 women die annually from preventable, pregnancy-related complications, with the majority of these deaths occurring in low-and-middle-income countries (LMICs) [1]. Proximity of households to comprehensive obstetric care, has been identified as a key determinant for preventing maternal mortality due to obstetric emergencies [2]. Comprehensive obstetric care also known as signal functions is defined as “life-saving care that defines a health facility with regards to its capacity to treat obstetric and newborn emergencies” [2]. These signal include: administration of parenteral antibiotics, parenteral anticonvulsants, parenteral uterotonics, removal of retained products, assisted vaginal delivery, manual removal of the placenta, resuscitation of the newborn, cesarean section and blood transfusion [2]. Essentially, if pregnant women can reach comprehensive obstetric facilities within a short-time period, then the chances of mortality due to pregnancy-related complications will be significantly reduced. However, findings on the association between proximity to facilities and facility utilization for delivery have been mixed. For instance, a recent meta-analysis of 31 studies in diverse settings showed that living within five kilometers of obstetric facilities was positively associated with facility delivery, and every one kilometer increase in travel time and distance was negatively associated with facility delivery [3]. This finding was applicable to settings such as Malawi and Zambia where the odds of facility delivery decreased by 65% and 27% for every ten-kilometer increase in distance to the closest facility. However, the same findings were not applicable in Uganda and Kenya where having access to facilities within three kilometers was not significantly associated with facility delivery [3–5], or in Burkina Faso [3, 6], where pregnant women who resided seven or more kilometers away were more likely to deliver at home.

More importantly, the increasing use of facilities for delivery care across LMICs in the past decades have not translated into the expected reductions in maternal mortality [7]. This suggests that the majority of facility deliveries likely occur in primary health facilities which have limited obstetric capacity, in contrast to higher-level facilities which are more likely to provide comprehensive obstetric care. For instance, a latent class analysis of health-seeking behaviors in Malawi showed that 66% of women utilize facilities that are closer to their households and offer free services, regardless of their obstetric capacity [8]. Only around one-third of women, with higher socio-economic status, were more likely to travel further distances from their households in search of better obstetric care [8–12]. In general, facilities that are proximal to households tend to be primary health facilities—which frequently lack skilled health providers, adequate infrastructure or signal functions, and have low volume of deliveries (making skills retention difficult) [7]. Thus, primary care facilities are poorly suited for managing obstetric emergencies that often arise during delivery. Taken together, proximity to facilities can only be effective in mitigating maternal mortality when such facilities are equipped to provide adequate obstetric care. Although several studies have examined the association between proximity to health facilities and facility delivery across LMICs, the majority have largely ignored the differences in obstetric capacities across these facilities and their impact on facility utilization for delivery [3, 5, 13–15]. Hence, an understanding of the distribution of comprehensive EmOC services within communities, and how this impacts facility utilization for delivery—is key for mitigating maternal mortality. The relationship between proximity to comprehensive obstetric care and facility utilization for delivery care is further complicated by the use of varied methods in measuring facility obstetric capacity [2, 16–18]. These different methods may misrepresent the real scenario of obstetric care availability within a service environment. We focus on two commonly used methods including: 1) the facility’s recent performance of signal functions only [2, 16], and 2) a composite index of obstetric care [17]. We hypothesize that depending on which method is used, the distribution of obstetric capacity (defined as comprehensive, basic, and less than basic obstetric capacity) within a service environment, may differ significantly, and this could result in disparate associations between proximity to comprehensive care and facility utilization for delivery care. Urban, or rural residence of women, may further modify the relationship between proximity to comprehensive obstetric care and facility utilization for delivery [3, 19, 20]. We investigate the joint effects of proximity with the two EmOC methods on place of delivery; and whether this relationship is modified by urban or rural residence, in two countries, Malawi, and Haiti. These countries were selected because they have the most recent data on facility delivery and obstetric care for LMICs. In addition, their health facility surveys are conducted as censuses of all health facilities in these countries allowing for linkage of health facility data to household-level data [21].

## Materials and methods

### Data sources

This cross-sectional study utilized linked publicly available data from the most recent surveys for health facility assessments from the Service Provision and Assessment (SPA) and household surveys from the Demographic Health Survey (DHS) for Malawi and Haiti. In particular, these were chosen nor only because they have recent SPA surveys conducted as a census of health facilities allowing for linkage with household surveys at the cluster level [21] but also they have high maternal mortality but different health care systems. For instance, Malawi has one of the highest maternal mortality rates in Africa (439 deaths per 100,000 births) and a high prevalence of facility-based delivery, with only about 9% of women delivering at home (ranging from 5% in urban settings to 12% in rural) [8]. Moreover, Haiti has the highest maternal mortality ratio in Latin America and the Caribbean (521 deaths per 100,000 live births) but also has a hierarchical and fragmented system of health care provision where larger, better-equipped facilities are concentrated in cities while smaller facilities are located in rural settings. [22, 23] The DHS, conducted every 5 years, is a nationally representative household-based survey of around 25,000 to 30,000 individuals that provides data on a wide range of indicators in the areas of population, health, and nutrition [24]. The SPA surveys provide a comprehensive overview of a country’s health service delivery. This includes nationally representative information on the performance of various types of health facilities providing maternal, child, and reproductive health services, as well as services for specific infectious diseases (such as sexually transmitted infections, HIV/AIDS, malaria and tuberculosis) [25]. The SPA surveys are designed as either censuses or samples of health facilities within countries and are implemented by the Ministries of Health. As part of the SPA, an average of eight providers per facility are interviewed to include providers for the services being assessed. The sample is comprised by those health providers present in the facility on the day of the assessment. [25]. The present study included data on women’s place of delivery from the DHS surveys for Malawi (2015–2016) and Haiti (2016–2017), as well as their SPA surveys: Malawi (2013–2014) and Haiti (2017–2018). The study population comprised women between the ages of 15 and 49 who had a childbirth in the past five years prior to the survey period in Malawi (n = 13,448 out of 17,286 survey participants) and Haiti (n = 5,005 out of 6,530). The data are geocoded to the household cluster level where women resided using unique identifiers. There were 850 clusters in Malawi with a range of 13–18 households per cluster. In Haiti, there were 450 clusters with a range of 8–14 households per cluster. The facilities included were not a sample, rather, household clusters were linked to all facilities providing delivery services (who had trained providers offering delivering services) in Malawi (n = 459 of 1060) and Haiti (n = 300 of 1033). For the linkage, we used spatial proximity analysis [26]. S1 Table shows the characteristics of the facilities providing delivery services in Malawi and Haiti. In addition, S2 Table shows the proportion of facilities within the specified distance measurements (5km, 10km, 15km) from the household clusters for Malawi and Haiti. An Institutional Review Board was not required as these constitute secondary data which are publicly available from the DHS website at dhsprogram.com.

### Measures

Exposure variable: The exposure was a classification of facilities based on a combination of proximity to comprehensive obstetric facilities and two EmOC assessment methods. The exposure was specified as follows:

Step 1: Two approaches to defining EmOC capacity including: 1) facility-reported performance of signal functions in the past 3 months, and 2) a composite index of obstetric care, were employed in classifying facilities into three levels of obstetric capacity including comprehensive, basic, or less than basic emergency obstetric EmOC for method 1; and high, medium, and low emergency obstetric capacity for method 2. The levels of obstetric capacity for method 1 have been established in the existing literature [2]. Notably, comprehensive EmOC facilities are those that report providing all nine signal functions in the past 3 months, basic EmOC facilities are those capable of providing seven out of the nine signal functions excluding blood transfusion and cesarean section, while ‘less than basic EmOC facilities’ provide less than seven signal functions (excluding blood transfusion and cesarean section) [2, 17, 19]. For method 2, We calculated the composite readiness score using an equal-weight approach. This method has been established as a preferred one for creating composite indices compared with other weighting schemes [27]. The composite index consisting of 53 indicators of obstetric care was developed by weighting each indicator as a fraction of the total number of items. Equal weight was assigned to the six domains of readiness (S3 Table) and to all indicators within the same domain; the sum of all domains was standardized to have a maximum of 100. Each facility’s score is then expressed as the percentage of the highest possible readiness for that facility [17, 27]. More details on the components and development of the composite index have been provided in prior literature [17]. To allow for comparisons between the composite index and EmOC method 1, cut points were selected to mirror the proportion of facilities providing comprehensive, basic, and less than basic EmOC capacity. They included 1) the 90^th^ percentile (high capacity); 2) 75^th^ to less than 90^th^ percentile (medium capacity); and 3) less than 75^th^ percentile (low capacity).

Step 2: Household clusters of eligible women were linked to obstetric facilities within 5km, 10km and 15km from their household clusters via spatial proximity analysis, a geo-spatial approach. Consistent with previous studies [19, 28], three threshold distances (5km, 10km and 15km) were selected for this study, exemplifying the probable distances that women travel to seek healthcare in various LMICs. The DHS routinely displaces the GPS coordinates of household clusters [up to 2km in urban and 5km in rural settings] as a measure to preserve the anonymity of respondents. However, our selected threshold distances accommodate the maximum displacement of households cluster coordinates by the DHS [27, 29]. The spatial proximity analysis dichotomized accessibility to facilities based on the aforementioned distances (5km, 10km, 15km) as access/no access. [30, 31].

Step 3: To assess the joint effects of the EmOC capacity of facilities (using the two different methods for defining EmOC capacity), a composite exposure variable was then specified by proximity of households to comprehensive or high-level facilities. The exposure variable comprised four mutually exclusive facility combinations and a reference group, with each combination accounting for the distribution of comprehensive facilities relative to basic facilities at specific distances (5km, 10km, 15km) from household clusters. The exposure categories included: 1) Comprehensive EmOC within 5 km; 2) Comprehensive EmOC within 10 kms (no comprehensive facility within 5km) + Basic EmOC/no Basic EmOC within 5 km; 3) Comprehensive EmOC within 15 kms (no comprehensive facility within 5km and 10km) + Basic EmOC within 5 or 10km; and 4) Comprehensive EmOC within 15 kms (no comprehensive facility within 5km and 10km) + no Basic EmOC within 5 or 10km). These categories were compared to a reference category consisting of permutations of no comprehensive facilities within 5km, 10km and 15km along with basic and less than basic EmOC facilities. The exposure categories were designed to account for the possible distribution of obstetric facilities that are proximal to a household cluster.

#### Outcome variable

Place of delivery indicates whether a woman delivered her most recent child (within the past five years preceding the survey) in a health facility or at home. Responses included a variety of categories including health centers, clinics, hospitals, dispensaries, respondent’s home, and traditional birth attendants. This variable was specified as a dichotomous variable: facility delivery vs non-facility delivery (home, traditional birth attendant).

#### Covariates

Consistent with previous studies, [17, 32] individual-level measures associated with proximity to care, and facility delivery were considered as covariates. They included maternal age, education, marital status, employment, region of residence, parity, birth order, religion, wealth index, type of union, autonomy in healthcare decision making, health insurance, religion, and use of antenatal services [31]. Facility characteristics including facility type, volume of deliveries, managing authority, number of facilities offering delivery services, were also presented, consistent with prior literature [19, 31]. Environmental characteristics including population density and annual precipitation were also assessed.

### Statistical analysis

Descriptive statistics were computed to characterize the selected individual and facility characteristics for Malawi and Haiti. The proportions of DHS clusters linked to comprehensive EmOC, basic EmOC or less than basic EmOC facilities were estimated. Bivariate analysis was conducted to examine the associations between selected covariates and place of delivery, as well as urban-rural differences in place of delivery. Multivariable log-binomial models were fitted to estimate the crude association between proximity to facilities and EmOC method with place of delivery. These models were adjusted for the minimal set of covariates (obtained using Principal Component Analysis) applicable to each country. The minimal set of covariates included in the PCA were those that were significant (P>0.05) in a bivariate analysis of each covariate and the outcome, in each country. Principal Component Analysis (PCA) was applied as a dimensionality reduction technique to allow the incorporation of a considerable number of covariates in the log-binomial models. The resulting principal components (independent combination of a set of variables which explain the maximum variability) were included in the models [33]. SAS PROC GENMOD was used for the regression analyses. Sample sizes presented in the tables are unweighted while proportions, prevalence ratios and 95% confidence intervals are weighted. Geospatial analysis was done using ArcMAP version 10.6. Statistical analysis was conducted using SAS v. 9.4.

## Results

### Characteristics of women who had a childbirth in the last 5 years by place of delivery, in Malawi and Haiti

In Malawi, women who delivered in facilities were mostly between the ages of 20 and 39 (84.4%), were married (77.9%), in monogamous unions (88%), had attained a primary education (65.2%), were employed (84.8%), belonged to the poorest wealth quintile (22.7%), and were predominantly Christian (84.8%; Table 1). Almost half of women reported that their healthcare decisions were jointly made with their husbands (48.7%). Over a third of women had three to four children (30%) and reported having on average at least four antenatal visits (52.1%; Table 1). Most of them resided in rural settings (85.9%), predominantly in the Southern region (46.1%). These characteristics were similar for women who had a facility delivery and those who did not. However, when compared with women who delivered in facilities, a greater proportion of women who delivered outside of facilities had no education (20.9% versus 11.8%) and belonged to the poorest quintile (33.7% versus 22.7%). Women who delivered outside of facilities were also likely to have 5 or more children (38.5%) and attend only 2–3 antenatal visits (50.0%; Table 1). The median population density in Malawi was 243.1 people per square km (IQR: 153.9–412.5) in the most recent survey year (2015) whereas the median annual precipitation was 72.4 millimeters (IQR: 61.9–83.8).

**Table 1:** Characteristics of women who had a childbirth in the last 5 years by place of delivery in Malawi and Haiti: Demographic Health Survey, 2015–2017.

	Malawi (N = 13448)			Haiti (N = 5005)		
DHS survey period	2015–2016			2016–2017		
	Facility (N = 12517)	Home/Other (N = 931)	P-value	Facility (N = 1961)	Home/Other (N = 3044)	P-value
**Individual characteristics**	% (SE)^a^^,^ ^b^			% (SE)		
Age categories			< .0001			0.0003
<20	8.9 (0.32)	6.1 (0.89)		4.3 (0.47)	5.8 (0.52)	
20–39	84.4 (0.41)	82.5 (1.31)		86.3 (0.89)	81.4 (0.77)	
40–49	6.8 (0.27)	11.4 (1.16)		9.4 (0.85)	12.8 (0.64)	
Marital status			0.02			< .0001
Married	77.9 (0.51)	73.3 (2.02)		66.2 (1.23)	73.2 (1.19)	
Unmarried	22.1 (0.51)	26.7(2.02)		33.8 (1.23)	26.8 (1.19)	
Type of union			0.001			0.12
Monogamous	88.0(0.47)	83.1(1.69)		88.8 (0.97)	86.8 (0.92)	
Polygamous	11.9(0.47)	16.9(1.69)		11.2 (0.97)	13.2 (0.92)	
Education			< .0001			< .0001
None	11.8 (0.48)	20.9 (1.73)		6.4 (0.65)	26.7 (1.46)	
Primary	65.2 (0.65)	70.1 (2.01)		28.0 (1.25)	44.7 (1.14)	
Secondary	20.9 (0.62)	8.8 (1.25)		57.3 (1.38)	27.7 (1.39)	
More than secondary	2.1 (0.45)	0.2 (0.16)		8.3 (0.84)	0.8 (0.22)	
Wealth quintile			< .0001			< .0001
Poorest	22.7 (0.61)	33.7 (2.00)		7.5 (0.72)	32.9 (1.99)	
Poorer	21.4 (0.56)	24.9 (1.88)		12.4 (1.13)	25.1 (1.37)	
Middle	19.1 (0.47)	20.7 (1.55)		22.4 (1.52)	20.6 (1.53)	
Richer	18.3 (0.55)	13.9 (1.45)		27.3 (1.45)	15.3 (1.20)	
Richest	18.4 (0.60)	6.6 (1.12)		30.4 (2.06)	6.0 (0.69)	
Employment			0.10			0.86
Employed	70.5 (0.67)	73.9 (1.98)		66.7 (1.46)	67.1 (1.22)	
Unemployed	29.5 (0.67)	26.0 (1.98)		33.3 (1.46)	32.9 (1.22)	
Religion			-			-
None	0.4 (0.09)	0.9 (0.42)		8.8 (1.14)	12.3 (1.06)	
Christian	84.8 (0.83)	85.6 (1.86)		89.9 (1.14)	86.1 (1.12)	
Voodooist				0.05 (0.05)	1.6 (0.32)	
Muslim	14.6 (0.82)	13.4 (1.81)				
Other	0.2 (0.05)	0				
Autonomy in healthcare decision making			0.04			0.01
Woman alone	18.1 (0.56)	22.5 (2.04)		30.1 (1.52)	24.4 (1.22)	
Husband alone	32.4 (0.67)	33.5 (2.19)		21.1 (1.15)	22.6 (1.05)	
Woman and husband	48.7 (0.87)	43.7 (2.13)		46.9 (1.66)	50.5 (1.42)	
Others	0.7 (0.11)	0.4 (0.24)		1.9 (0.39)	2.5 (0.39)	
Health insurance			0.04			< .0001
Yes	1.4 (0.34)	0.27 (0.24)		5.2 (0.65)	0.48 (0.15)	
No	98.6 (0.34)	99.7 (0.24)		94.8 (0.65)	99.5 (0.15)	
Parity			< .0001			< .0001
1–2 children	45.9 (0.64)	30.6 (1.91)		68.0 (1.30)	44.7 (1.16)	
3–4 children	30.0 (0.51)	30.9 (1.91)		20.6 (1.12)	28.6 (1.12)	
> = 5 children	24.1 (0.52)	38.5 (1.79)		11.3 (0.96)	26.7 (1.11)	
Birth order			< .0001			< .0001
1	25.2 (0.52)	13.1 (1.33)		43.3 (1.34)	21.2 (0.88)	
2–3	37.3 (0.57)	32.8 (1.86)		37.4 (1.41)	40.3 (1.15)	
4–5	23.6 (0.49)	26.9 (1.92)		13.4 (0.98)	19.7 (0.86)	
6+	13.9 (0.39)	27.1 (1.83)		5.9 (0.64)	18.9 (0.99)	
Antenatal care visits			< .0001			< .0001
None	1.2 (0.18)	9.2 (1.11)		2.4 (0.45)	13.1 (0.94)	
1	1.8 (0.16)	5.4 (0.89)		2.0 (0.39)	6.5 (0.59)	
2–3	44.9 (0.66)	50.9 (1.96)		13.3 (0.89)	25.0 (0.94)	
4+	52.1 (0.68)	34.4 (1.96)		82.3 (1.09)	55.4 (1.49)	
**Contextual characteristics**						
Comprehensive EmOC			0.01			< .0001
Within 5km	12.7 (1.22)	5.5 (1.07)		40.5 (3.15)	19.7 (2.28)	
10km+basic/no basic EmOC within 5km	14.7 (1.59)	13.3 (2.64)		17.9 (2.62)	15.2 (2.15)	
15km + basic EmOC within 5	2.9 (0.84)	6.4 (2.85)		6.0 (1.63)	6.0 (1.49)	
15km + no basic EmOC within 5	8.9 (1.25)	9.8 (2.79)		6.5 (1.46)	11.9 (2.35)	
No comp EmOC	60.9 (1.77)	64.9 (3.85)		29.1 (2.41)	47.1 (2.99)	
Residence			0.001			< .0001
Urban	15.0 (0.50)	6.6 (1.61)		53.9(2.14)	25.9(1.76)	
Rural	85.9 (0.50)	93.4 (1.61)		46.1 (2.14)	74.1(1.76)	
Region^c^			< .0001			< .0001
North	11.7 (0.39)	11.3 (1.55)				
Central	42.1 (0.69)	43.7 (3.03)				
South	46.1 (0.67)	44.9 (2.95)				
Aire-metropolitaine	-	-		27.9 (1.77)	13.7 (1.63)	
Rest-quest	-	-		15.5 (1.89)	20.4 (1.92)	
Sud-Est	-	-		3.6 (0.49)	6.0 (0.99)	
Nord	-	-		10.8 (1.54)	11.2 (0.96)	
Nord-Est	-	-		4.1 (0.51)	3.8 (0.45)	
Artibonite	-	-		13.8 (1.84)	17.1 (1.41)	
Center	-	-		8.6 (0.97)	7.2 (0.76)	
Sud	-	-		6.1 (0.75)	6.7 (0.74)	
Grand’ anse	-	-		2.9 (0.49)	5.4 (0.67)	
Nord-Ouest	-	-		3.8 (0.48)	5.9 (0.48)	
Nippes	-	-		2.9 (0.45)	2.6 (0.29)	
**Environmental characteristics**						
Annual precipitation Median (IQR)	72.4 (61.9–83.8)	72.4 (61.9–83.8)	0.33	101.7 (93.2–105.9)	101.7 (93.2–120.3)	0.001
Population density Median (IQR)	243.1 (153.9–412.5)	229.3 (144.7–349.1)	< .0001	498.1 (268.8–2833.8)	298.5 (208.9–533.2)	< .0001

^a^Weighted percentages are shown, with corresponding standard errors (SE).

^b^Percentages may not add up to 100 as a result of rounding.

^c^The regions applicable to Malawi (North, Central and South) differ from that of Haiti.

In Haiti, the majority of women who had a facility delivery were also between the ages of 20 and 39 (86.3%), mostly married (77.9%), in monogamous unions (88.8%), had obtained a secondary education (57.3%), were employed (66.7%), belonged to the poorest quintile (32.9%) and were mostly Christian (89.9%). Almost half of these women reported that their healthcare decisions were jointly made with their husbands (46.9%). Most of them had at least one child and reported having at least 4 antenatal visits (52.1%). Almost one-third of these women resided in urban settings, in the Aire Metropolitaine region (27.9%). When compared with women who had a facility delivery, a greater proportion of women who delivered outside of facilities had no education (26.7% versus 6.4%), belonged to the poorest quintile (32.9% versus 7.5%), have 5 or more children (26.7% versus 11.3%), and attend only 2–3 antenatal visits. The median population density (498.1 people per square km, IQR = 268.8–2833.8) and annual precipitation (101.7 millimeters, IQR: 93.2–105.9) were considerably higher in Haiti than Malawi.

There were no differences in prevalence of facility delivery by employment status for both Malawi and Haiti (Table 1). However, as seen in Table 1, facility delivery was associated with type of marital union in Malawi but not in Haiti, and for annual precipitation in Haiti but not in Malawi. Additionally, facility delivery was associated in both countries with age groups, marital status, wealth quintile, autonomy in decision-making, health insurance, parity, birth order, antenatal visits, residence, and population density (p-values <0.05) (Table 1).

### Association between the joint effect of proximity and EmOC methods with place of delivery, in Malawi and Haiti

In Malawi, proximity was associated with place of delivery only among women living within 5km from a comprehensive facility, across both EmOC methods (Table 2). However, in the adjusted analysis, the association remained only in EmOC method 2 (Table 3). For Haiti, the likelihood of delivering in a comprehensive EmOC facility was higher with greater proximity of facilities to households, regardless of EmOC method (Table 2).

**Table 2:** Crude association between proximity to obstetric care on facility delivery among women residing in Malawi and Haiti using two EmOC assessment methods: Demographic Health Surveys and Service Provision Assessments 2014–2018.

Method, Prevalence Ratio (95% CI)	Malawi	Haiti
EmOC 1^a^^,^ ^b^		
Comprehensive EmOC within 5km	1.03 (1.01,1.06)	2.14 (1.82,2.53)
Comprehensive EmOC within 10 km + Basic/no Basic EmOC within 5 km	1.02 (0.99,1.05)	1.63 (1.35,1.97)
Comprehensive EmOC within 15 km + Basic EmOC within 5 or 10km	0.94 (0.78,1.14)	1.52 (1.17,2.03)
Comprehensive EmOC within 15 km + no Basic EmOC within 5 or 10km	0.97 (0.92,1.02)	1.13 (0.81,1.58)
EmOC 2		
Comprehensive EmOC within 5km	1.05 (1.03,1.07)	1.93 (1.65,2.27)
Comprehensive EmOC within 10 km + Basic/no Basic EmOC within 5 km	1.01 (0.98,1.04)	1.49 (1.22,1.81)
Comprehensive EmOC within 15 km + Basic EmOC within 5 or 10km	0.92 (0.79,1.06)	1.36 (1.05,1.76)
Comprehensive EmOC within 15 km + no Basic EmOC within 5 or 10km	0.99 (0.95,1.04)	0.91 (0.70,1.17)

^a^Reference group: no comprehensive EmOC within 5, 10 or 15km + combination of Basic/less than Basic EmOC facilities.

^b^All groups are mutually exclusive (e.g. comprehensive EmOC within 10km implies no comp facility within 5km).

**Table 3:** Adjusted association between proximity to obstetric care on facility utilization for delivery care among women in Malawi and Haiti using two EmOC assessment methods: Demographic Health Surveys and Service Provision Assessments 2014–2018.

Method, Prevalence Ratio (95% CI)	Malawi^c^	Haiti
EmOC 1^a^^,^ ^b^		
Comprehensive EmOC within 5km	1.01 (0.99,1.04)	1.81 (1.56,2.09)
Comprehensive EmOC within 10 km + Basic/no Basic EmOC within 5 km	1.01 (0.99,1.04)	1.45 (1.22,1.71)
Comprehensive EmOC within 15 km + Basic EmOC within 5 or 10km	0.92 (0.75,1.12)	1.44 (1.15,1.82)
Comprehensive EmOC within 15 km + no Basic EmOC within 5 or 10km	0.96 (0.91,1.02)	1.12 (0.83,1.50)
EmOC 2		
Comprehensive EmOC within 5km	1.03 (1.01,1.05)	1.27 (1.12,1.44)
Comprehensive EmOC within 10 km + Basic/no Basic EmOC within 5 km	1.02 (0.99,1.04)	1.14 (0.98,1.34)
Comprehensive EmOC within 15 km + Basic EmOC within 5 or 10km	0.90 (0.79,1.04)	1.24 (1.03,1.48)
Comprehensive EmOC within 15 km + no Basic EmOC within 5 or 10km	0.99 (0.96,1.04)	0.92 (0.77,1.11)

^a^Reference group: no comprehensive EmOC within 5, 10 or 15km + combination of Basic/less than Basic EmOC facilities.

^b^All groups are mutually exclusive (e.g. comprehensive EmOC within 10km implies no comprehensive facility within 5km).

^c^The models for Malawi and Haiti were adjusted for maternal age, wealth quintile, religion, occupation, marital status, health insurance, birth order and antenatal visits. In addition, the model for Malawi was adjusted for marital status and annual precipitation, while that of Haiti was adjusted for region and autonomy in healthcare decision-making.

For Haiti, women living within 5km of a comprehensive EmOC facility were more likely to deliver in a facility, based on EmOC method 1 (APR: 1.81, 95% CI 1.56, 2.09) and EmOC method 2 (APR: 1.27, 95% CI 1.12, 1.44; Table 3) after adjustment. However, women living within 10km of a comprehensive EmOC facility were more likely to deliver in a facility (APR: 1.44, 95% CI 1.15, 1.82), regardless of whether a basic EmOC facility was closer to them—based on EmOC method 1 only. The association for women living within 10km of comprehensive facilities in Haiti was not significant for EmOC method 2 (APR 1.12, 95%CI 0.83, 1.50). Among women living within 15km of a comprehensive EmOC facility, those that had at least one basic EmOC facility within 5km or 10km were more likely to deliver in a facility—-based on both EmOC method 1 (APR: 1.27, 95% CI 1.12, 1.44) and method 2 (APR: 1.24, 95% CI 1.03, 1.48). However, these associations were not significant for women who lived within 15km of a comprehensive facility, and did not have any basic facility closer to them, were less likely to have a facility delivery—regardless of EmOC methods.

### Urban-rural differences in the association between proximity to comprehensive obstetric care and place of delivery in Malawi and Haiti

For Malawi, proximity to comprehensive care was significantly associated with facility delivery in urban settings (for EmOC 1 only) but not in rural settings (Table 4). The association was not significant for EmOC method 2. Specifically, living within 15km of a comprehensive facility (whether a basic facility was within 5km or not) was significantly associated with a lower probability of facility utilization for delivery (APR: 0.53, 95% CI 0.28, 0.98), based on EmOC method 1. For Haiti, the association was significant and varied by proximity, EmOC method, and urban/rural setting. The association was stronger and positive in general for rural settings compared with urban settings where associations were under or close to 1.00. In addition, the likelihood of delivering in a comprehensive EmOC facility in rural settings was higher with greater proximity of facilities to households, regardless of EmOC method.

**Table 4:** Urban-rural differences in the association between proximity to obstetric care on place of delivery among women in Malawi and Haiti using two EmOC assessment methods: 2014–2018 DHS and SPA surveys.

	Malawi^c^		Haiti	
Method, Prevalence Ratio (95% CI)	Urban	Rural	Urban	Rural
EmOC 1^a^^,^ ^b^				
Comprehensive EmOC within 5km	1.00 (0.98, 1.02)	0.97 (0.91,1.03)	0.94 (0.83,1.07)	1.61 (1.36,1.91)
Comprehensive EmOC within 10 km + Basic/no Basic EmOC within 5 km	0.99 (0.96, 1.02)	1.01 (0.98,1.04)	0.81 (0.68,0.96)	1.36 (1.10,1.68)
Comprehensive EmOC within 15 km + Basic EmOC within 5 or 10km^d^	0.53 (0.28, 0.98)	1.01 (0.96,1.07)	0.99 (0.79,1.26)	1.50 (1.19,1.89)
Comprehensive EmOC within 15 km + no Basic EmOC within 5 or 10km	-	0.96 (0.91,1.02)	0.74 (0.62,0.88)	1.28 (1.01,1.63)
EmOC 2^e^				
Comprehensive EmOC within 5km	0.99 (0.97, 1.02)	1.02 (0.98,1.05)	1.05 (0.91,1.19)	1.35 (1.13,1.62)
Comprehensive EmOC within 10 km + Basic/no Basic EmOC within 5 km	0.97 (0.90, 1.04)	1.02 (0.99,1.04)	0.90 (0.73,1.11)	1.31 (1.08,1.58)
Comprehensive EmOC within 15 km + Basic EmOC within 5 or 10km	1.02 (0.97, 1.04)	0.90 (0.78,1.04)	1.08 (0.78,1.49)	1.28 (1.03,1.58)
Comprehensive EmOC within 15 km + no Basic EmOC within 5 or 10km	-	0.99 (0.96,1.04)	0.87 (0.68,1.10)	0.96 (0.77,1.21)

^a^Reference group: no comprehensive EmOC within 5, 10 or 15km + combination of Basic/less than Basic EmOC facilities.

^b^All groups are mutually exclusive (e.g. comprehensive EmOC within 10km implies no comprehensive facility within 5km).

^c^The models for Malawi and Haiti were adjusted for maternal age, wealth quintile, religion, occupation, marital status, health insurance, birth order and antenatal visits. In addition, the model for Malawi was adjusted for marital status and annual precipitation, while that of Haiti was adjusted for region and autonomy in healthcare decision-making.

^d^Categories 3 and 4 for EmOC 1 were combined for urban settings in Malawi due to small cell counts (that prevented convergence) in category 3, hence the estimates are the same.

^e^There were no records of women belonging to Category 4 of EMOC 2, hence no estimates were generated.

## Discussion

The findings showed that in Malawi, proximity to comprehensive care was only significantly associated with facility utilization for delivery among women living 5km from their households (using EmOC method 2), however, when stratifying by urban/rural setting, proximity to facilities was significantly associated with lower likelihood of facility delivery in urban settings only among women living 15km of comprehensive facilities (based on EmOC method 1 only). In rural settings of Malawi, the association was not significant regardless of EmOC method. In Haiti, the likelihood of utilizing a comprehensive EmOC facility was higher with greater proximity of facilities to households, regardless of EmOC method. Proximity to comprehensive obstetric facilities remained significantly associated with facility delivery in the adjusted analysis for Haiti, but mostly for EmOC method 1. Regardless of EmOC method, living within 5km of a comprehensive EmOC facility was significantly associated with a greater likelihood of facility delivery in Haiti. In addition, living within 15km of a comprehensive EmOC facility was significantly associated with a greater likelihood of facility delivery in Haiti, only when a basic EmOC facility was available within 5km or 10km. However, based on EmOC method 1 alone, living within 10km of a comprehensive EmOC facility in Haiti was significantly associated with a greater likelihood of facility delivery, given that there was at least a basic facility within 5km. The magnitude of association was stronger in rural compared with urban settings of Haiti.

Our findings showed that proximity to facilities was significantly associated with place of delivery and further this association varied by EmOC method, in Haiti. Although prior studies have largely investigated the association between proximity to facilities and place of delivery, our study is among a few that have explored EmOC categorizations in relation to facility proximity. The direction of association in prior studies was similar to ours. For instance, a recent meta-analysis showed that living within 5km of a facility was associated with a greater likelihood of delivering in a facility (OR = 2.27, 95% CI 1.82, 2.82), [3] similar to the present study’s findings for Haiti (APR: 1.81, 95% CI 1.56, 2.09). Notably, the odds ratio tends to overestimate the prevalence ratio when the outcome is common—this could explain the differences in the adjusted OR from the prior study and the present study’s APR. Our finding in Haiti showing that—living within 10km or 15km of a comprehensive facility was associated with facility delivery only when there was a basic facility closer to the household—suggests that women may have a stronger preference for facilities that are closer to their households, even if these facilities do not have a comprehensive EmOC capacity. This suggestion is consistent with findings from a recent latent class analysis of social determinants of health-seeking behavior among pregnant women in Malawi [8]. Findings from that study showed that the majority of women prefer to use facilities that are closer and offer free services, regardless of their quality [8]. Additionally, physical accessibility remains a huge barrier to facility utilization in Haiti, owing to its mountainous terrain [19]. Such accessibility challenges further support women’s use of facilities that are closer to their households. Overall, the strength of association in Haiti was stronger for EmOC method 1, regardless of proximity to facilities. In addition, our finding showing that the association [between proximity to a comprehensive facility within 10km and place of delivery] in Haiti was present only for EmOC method 1 suggests that how we measure and define comprehensive EmOC matters. These differences across methods may be attributed to their components—EmOC method 1 may be viewed as a less conservative approach since it considers only signal functions in assessing facility obstetric capacity, whereas EmOC method 2 combines a set of 53 facility indicators [18], making it more conservative. Thus, since a fewer number of facilities will likely have all 53 indicators, the more conservative approach (EmOC method 2) might exhibit a weaker association with the outcome. The positive associations identified suggest that proximity to a health facility and most foremost, quality of care provided at health facilities plays important roles in the use of services in Haiti. Notably, access to comprehensive delivery services in Haiti has been largely impacted by the destruction of health facilities in the 2010 earthquake, coupled with the mountainous terrain especially in rural areas [23, 34]. In Haiti, a large number of facilities have limited capacity to provide comprehensive obstetric care with most of the facilities lacking essential delivery care equipment and supplies. For instance, in Haiti, only about 30% of facilities have functional emergency transportation, staff do not receive regular training in comprehensive obstetric care, and guidelines for providing such care are frequently absent in most facilities [22, 35], all of which increase the risk of maternal and newborn mortality.

Our findings showing a stronger association between proximity and place of delivery in rural settings (compared with urban settings) in Haiti, was consistent with a prior DHS study [19]. The DHS study examined the association between proximity to obstetric care and facility delivery in Haiti (using the 2012 and 2013 DHS and SPA surveys) [19]. In that study, obstetric care was characterized using a composite approach in the prior study, similar to EmOC method 2 in our study. The prior study found that women living within 10km of high-capacity obstetric facilities in rural settings were 1.5 times likely to deliver in facilities (AOR: 1.53, 95% CI 1.29, 1.80), whereas the association was not significant in urban settings (AOR: 1.36, 95% CI 1.01, 1.84) [19]. This finding was similar to our study which showed a greater likelihood of facility delivery among women living within 10km of comprehensive EmOC facilities in rural settings of Haiti (APR: 1.31, 95% CI 1.08, 1.58) but not in urban settings (APR: 0.90, 95% CI 0.73, 1.11), based on EmOC method 2.

Our study finding showed that the association between proximity and place of delivery was only significant in Malawi, among women living within 5km of comprehensive facilities (using EmOC method 2), and in urban settings among women living within 15km of comprehensive facilities. We partly attribute our findings in Malawi to the regression approach employed (log-binomial) which is a more conservative analysis than logistic regression, a more commonly utilized approach. However, we mostly attribute the null and lower likelihood to the universal coverage of facility delivery in Malawi (92%), enforced by national policies prohibiting the practices of traditional birth attendants who conduct home-deliveries [8]. This is in contrast to Haiti where facility delivery coverage is about 42% (as estimated in our study). The much higher coverage rates in Malawi may explain the negligible effect of facility type (EmOC method) or the adjusted covariates. In addition, proximity to care has been shown to play a greater role in pregnant women’s choice to utilize facility delivery, compared with structural quality of care in Malawi [8]. Evidence shows that majority of women in Malawi seem to have a higher preference for facilities that do not charge user fees, while only a smaller fraction of women (older aged with higher socioeconomic status) prioritize facilities with better obstetric care [8]. This suggests that there may be limited interest in seeking/utilizing comprehensive obstetric care among the majority of women in Malawi. The latter could partly contribute to the negligible association found between proximity to comprehensive care and facility utilization for delivery in Malawi.

The present study had several limitations. The self-reported nature of the SPA data could overestimate the measures of association since health facilities may report having better EmOC capacity, and prior studies have shown that women intentionally seek out facilities with better capacity. The measures of association from the present study also need to be interpreted with caution. Notably, the DHS data only assessed if women reported having a facility delivery and did not directly measure the types or location of facilities (comprehensive, basic, less than basic EmOC) they utilized. Hence, the geographic linkage of these facility categories to women in household clusters may imply that women utilized those specific facilities which may not be the case, since geographic access (distance to facility) does not equal utilization. In addition, the differences in methodology for the SPA surveys across countries further limits the ability to link most country surveys with their corresponding DHS household surveys. In particular, Malawi and Haiti were the only countries with recent SPA surveys conducted as a census of health facilities, and thus, allow for linkage with household surveys at the cluster level [21]. Most health facility surveys are conducted as a sample of facilities in the countries thus limiting geographic linkage of the SPA survey with their corresponding household surveys (DHS). Despite these limitations, the present study has several strengths. Our findings concur with prior findings that women tend to bypass facilities in search of better obstetric care, emphasizing that in addition to facility obstetric capacity, the method through which obstetric capacity is determined is pertinent [9–11, 36, 37]. In addition, our study elucidated the relevance of context in the association under study. In particular, high facility coverage rates could largely nullify the relationship between proximity and facility utilization, as seen in the case of Malawi. Another important strength of this study is the use of log-binomial regressions (which yield more conservative estimates) as opposed to logistic regressions (that tend to overestimate estimates for common outcomes) which have been frequently used in similar studies.

## Conclusions

This study demonstrates the critical need to consider how best to measure EmOC capacity and to include availability of comprehensive EmOC when examining the relationship between proximity to clinics and facility delivery. The findings contribute to the obstetric care literature, by being among the first to examine the joint effect of proximity to clinics and level of EmOC capacity available on place of delivery. While a variety of studies have examined the relationship between proximity to facilities and facility utilization for delivery, a key innovation of this study is the consideration of the facility’s capacity to provide comprehensive emergency obstetric care, and impact of using multiple methods [to characterize comprehensive emergency obstetric care] on facility utilization. Such findings could potentially inform policy and intervention efforts in relation to geolocation of obstetric facilities in diverse settings. The finding in Haiti showing that—living within 10km or 15km of a comprehensive facility was associated with a greater likelihood of facility delivery only when there was a basic facility closer to the household—suggests that women may have a stronger preference for facilities that are closer to their households, even if these facilities do not provide comprehensive EmOC. Hence, interventions to increase women’s utilization of comprehensive EmOC facilities should not only include improvements in health system and transportation infrastructure, but also educating women on the relevance of seeking better obstetric care, even if they have to travel a greater distance to obtain it. In light of the variation in findings by how EmOC capacity was measured, greater work on validating how best to measure this capacity is needed, which also allow for different contexts and tools. In addition, the present study’s finding of null associations for Malawi are mostly attributed to the high rates of facility delivery in the country (92%), which makes the effect of geographic access and methods negligible. This underscores the relevance of context (high facility delivery rates, in this case) when examining the relationship between geographic access and facility delivery—which craves further investigation.

## Supporting information

S1 Table Background characteristics of health facilities offering delivery services and having trained providers who offer delivery or newborn services in Malawi and Haiti: 2013–2018 SPA. (DOCX)

S2 Table Percentage of household clusters having comprehensive, basic, and less than basic facilities within 5km, 10km and 15km, in Malawi and Haiti: 2014–2018 DHS and SPA surveys. (DOCX)

S3 Table Measures included in the composite index defined by the Demographic Health Survey program in August 2018. (DOCX)
