# Supplementary material for: Effect of emergency obstetric care and proximity to comprehensive facilities on facility-based delivery in Malawi and Haiti
Source: PLOS Glob Public Health. 2022 Feb 2;2(2):e0000184. doi: 10.1371/journal.pgph.0000184 (PMC10021570; doi:10.1371/journal.pgph.0000184)
Supplement: S1 Table — (DOCX) [file pgph.0000184.s001.docx]

**S1 Table. Background characteristics of health facilities offering delivery services and having trained providers who offer delivery or newborn services in Malawi and Haiti: 2013-2018 SPA**

|  | **Malawi** | | | | **Haiti** | | | |
| --- | --- | --- | --- | --- | --- | --- | --- | --- |
| **SPA survey period** | 2013-2014 | | | | 2017-2018 | | | |
|  | **Total**  **(N=459)** | **Urban**  **(N=76)** | **Rural**  **(N=383)** | **P-value** | **Total**  **(N=300)** | **Urban**  **(N=128)** | **Rural**  **(N=172)** | **P-value** |
| Managing authority |  |  |  | 0.01 |  |  |  | 0.0004 |
| Public | 67.8 (1.94) | 57.2 (4.59) | 70.5 (2.28) |  | 44.6 (2.73) | 52.2 (4.12) | 38.9 (3.66) |  |
| Private | 32.2 (1.94) | 42.8 (4.59) | 29.5 (2.28) |  | 37.8(2.61) | 39.9 (4.01) | 36.1 (3.62) |  |
| Mixed |  |  |  |  | 17.6 (2.17) | 7.8 (2.36) | 24.9 (3.29) |  |
| Facility type |  |  |  | - |  |  |  | <.0001 |
| Hospital | 25.3 (0.60) | 67.7 (4.6) | 14.5 (1.11) |  | 33.3 (0.04) | 55.4 (3.07) | 16.9 (2.23) |  |
| Health center | 71.4 (0.62) | 26.9 (4.6) | 82.8 (1.16) |  | 50.7 (0.05) | 43.1 (3.08) | 56.3 (2.15) |  |
| Dispensary |  |  |  |  | 16.0 (0.02) | 1.6 (1.08) | 1.6 (1.08) |  |
| Clinic | 2.5 (0.11) | 5.5 (1.94) | 1.8 (0.45) |  |  |  |  |  |
| Maternity | 0.7 (0.17) | - | 0.9 (0.22) |  |  |  |  |  |
| Region |  |  |  | 0.19 |  |  |  | <.0001 |
| North | 18.6 (1.81) | 18.7 (4.51) | 18.6 (1.97) |  |  |  |  |  |
| Central | 39.8 (2.44) | 48.3 (6.28) | 37.6 (2.58) |  |  |  |  |  |
| South | 41.6 (2.45) | 33.1 (5.55) | 43.8 (2.69) |  |  |  |  |  |
| Ouest | - | - | - |  | 29.7 (2.49) | 46.8 (4.22) | 17.0 (2.87) |  |
| Sud-est | - | - | - |  | 9.9 (1.72) | 3.9 (1.72) | 14.5 (2.69) |  |
| Nord | - | - | - |  | 10.6 (1.77) | 10.9 (2.79) | 10.4 (2.29) |  |
| Nord-est | - | - | - |  | 5.3 (1.29) | 6.2 (2.14) | 4.6 (1.59) |  |
| Artibonite | - | - | - |  | 9.6 (1.71) | 8.6 (2.48) | 10.4 (2.34) |  |
| Center | - | - | - |  | 6.9 (1.47) | 6.2 (2.17) | 7.5 (2.01) |  |
| Sud | - | - | - |  | 6.9 (1.47) | 7.0 (2.26) | 6.9 (1.93) |  |
| Grand’anse | - | - | - |  | 5.3 (1.29) | 2.3 (1.34) | 7.5 (2.02) |  |
| Nord-ouest | - | - | - |  | 12.5 (1.78) | 4.9 (1.96) | 18.2 (2.75) |  |
| Nippes | - | - | - |  | 2.9 (0.98) | 3.1 (1.55) | 2.9 (1.27) |  |
| 24-hour coverage of health facilities by providers |  |  |  | <.0001 |  |  |  | 0.001 |
| Yes | 56.3 (2.26) | 83.1 (4.87) | 49.9 (2.61) |  | 53.3 (2.63) | 64.9 (4.04) | 44.7 (3.62) |  |
| No | 43.7 (2.26) | 16.9 (4.87) | 50.1 (2.61) |  | 46.7 (2.63) | 35.1 (4.04) | 55.3 (3.62) |  |
| Average facility delivery recorded among providers in the past 6 months |  |  |  |  |  |  |  | 0.001 |
| (Median, IQR) | 80.0 (39.3-153.3) | 50.0 (17.0-112.7) | 86.5 (40.5-162.6) | 0.2813 | 10.0 (4.0-30.0) | 20.0 (7.3-40.4) | 7.8 (3.0-16.8) |  |

*The regions applicable to Malawi (North, Central and South) differ from that of Haiti
