# Supplementary material for: Effect of emergency obstetric care and proximity to comprehensive facilities on facility-based delivery in Malawi and Haiti
Source: PLOS Glob Public Health. 2022 Feb 2;2(2):e0000184. doi: 10.1371/journal.pgph.0000184 (PMC10021570; doi:10.1371/journal.pgph.0000184)
Supplement: S2 Table — (DOCX) [file pgph.0000184.s002.docx]

**S2.** **Percentage of household clusters having comprehensive, basic and less than basic facilities within 5km, 10km and 15km, in Malawi and Haiti: 2014-2018 DHS and SPA surveys**

|  | **Malawi (Total clusters N=850)** | | | **Haiti (Total clusters N=450)** | | |
| --- | --- | --- | --- | --- | --- | --- |
| **Methods, Percentages (%, N)** | 5km | 10km | 15km | 5km | 10km | 15km |
| **EmOC Method 1** |  |  |  |  |  |  |
| Comprehensive facilities (total) | 17.3 (147) | 27.9 (237) | 38.7 (329) | 25.3 (114) | 42.9 (193) | 55.3 (249) |
| Average access | 17.3 (147) | 27.9(237) | 3.4 (29) | 25.3 (114) | 7.6 (34) | 20.6 (93) |
| Most access |  |  | 35.3 (300) |  | 35.3 (159) | 34.6 (156) |
| Basic facilities (total) | 4.7 (40) | 13.9 (118) | 25.1 (213) | 8.9 (40) | 29.1 (131) | 53.8 (242) |
| Average access | 4.7 (40) | 13.9 (118) | 25.1 (213) | 8.9 (40) | 29.1 (131) | 20.7 (93) |
| Most access |  |  |  |  |  | 33.1 (149) |
| Less than basic facilities (total) | 47.1 (400) | 66 (561) | 64.8 (551) | 55.5 (250) | 67.1 (302) | 67.6 (304) |
| Average access | 13.5 (115) | 33.6 (286) | 32.7 (278) | 20.4 (92) | 34.0 (153) | 33.8 (152) |
| Most access | 33.5 (285) | 32.4 (275) | 32.1 (273) | 35.1 (158) | 33.1 (149) | 33.8 (152) |
| **EmOC Method 2** |  |  |  |  |  |  |
| Comprehensive facilities (total) | 15.3 (130) | 26.4 (224) | 36.4 (309) | 22.0 (99) | 38.7 (154) | 54.2 (244) |
| Average access | 15.3 (130) | 26.4 (224) | 0.9 (8) | 22.0 (99) | 3.8 (17) | 20.7 (93) |
| Most access |  |  | 35.4 (301) |  | 34.9 (157) | 33.6 (151) |
| Basic facilities (total) | 99.5 (846) | 99.5 (846) | 47.8 (406) | 27.6 (124) | 51.3 (231) | 67.3 (303) |
| Average access | 82.2 (699) | 68.2 (580) | 13.3 (113) | 27.6 (124) | 16.7 (75) | 34.4 (155) |
| Most access | 17.3 (147) | 31.3 (266) | 34.5 (293) |  | 34.7 (156) | 32.9 (148) |
| Less than basic facilities (total) | 39.5 (336) | 65.2 (850) | 64.4 (547) | 56.7 (255) | 67.3 (303) | 66 (297) |
| Average access | 6.7 (57) | 33.5 (285) | 32.7 (278) | 21.6 (97) | 33.6 (151) | 32.7 (147) |
| Most access | 32.9 (279) | 31.8 (270) | 31.6 (269) | 35.1 (158) | 33.8 (152) | 33.3 (150) |

*Kernel density estimation was employed in characterizing access (average access and most access
