# Supplementary material for: Effect of emergency obstetric care and proximity to comprehensive facilities on facility-based delivery in Malawi and Haiti
Source: PLOS Glob Public Health. 2022 Feb 2;2(2):e0000184. doi: 10.1371/journal.pgph.0000184 (PMC10021570; doi:10.1371/journal.pgph.0000184)
Supplement: S3 Table — (DOCX) [file pgph.0000184.s003.docx]

**S3 Table. Measures included in the composite index defined by the Demographic Health Survey program in August 2018**

| **Domain/Indicator name** | **Definition** |
| --- | --- |
|  |  |
| **Domain A: Comprehensive emergency obstetric care** | |
| Parenteral administration of antibiotics | Facility provided this signal function at least once in the past three months |
| Parenteral administration of uterotonics | Same as above |
| Parenteral administration of anticonvulsants for pregnancy-related hypertensive disorders | Same as above |
| Manual removal of placenta | Same as above |
| Removal of retained products of conception | Same as above |
| Assisted vaginal delivery | Same as above |
| Basic neonatal resuscitation | Same as above |
| Cesarean section | Same as above |
| Blood transfusion | Same as above |
| **Domain B: Newborn signal functions and immediate care** | |
| Neonatal resuscitation | Facility provided this signal function at least once in the past three months |
| Skin to skin | Facility reported that this intervention is practiced routinely |
| Breastfeeding in first hour | Same as above |
| Drying and wrapping newborns | Same as above |
| **Domain C: General requirements** | |
| Electricity | Connection to a central power grid without interruption in power supply lasting for more than two hours at a time during normal working hours in the seven days prior to the assessment / presence of a functioning generator with fuel available on the day of the assessment /Presence of a backup solar power |
| Improved water source | Improved water source available. For most countries, this is equivalent to water piped into the facility or onto facility grounds/ public tap or standpipe water source/ tube well or borehole/ protected dug well /protected spring /rainwater/bottled water, and the outlet from this source is within 500 meters of the facility. |
| Improved sanitation | Facility has a functioning flush or pour-flush toilet/ventilated improved pit latrine/composting toilet. |
| 24/7 skilled birth attendance | Provider of delivery care available on-site or on-call 24 hours a day, with observed duty schedule |
| Emergency transport | The facility had a functioning ambulance or other vehicle for emergency transport that was stationed at the facility and had fuel available on the day of the assessment, or the facility has access to an ambulance or other vehicle for emergency transport that is stationed at another facility or that operates from another facility |
| **Domain D: Equipment** | |
| Sterilization equipment | Facility reports that some instruments are processed in the facility and the facility has a functioning electric dry heat sterilizer, a functioning electric autoclave, or a non-electric autoclave with a functioning heat source available somewhere in the facility |
| Delivery bed | At least one delivery bed available and observed in delivery area. |
| Examination light | Examination light (flashlight okay) available, observed, and functioning in delivery area. |
| Delivery pack | Delivery pack OR cord clamp, episiotomy scissors, scissors/lade to cut cord, suture material with need, AND needle holder all available in delivery area. |
| Suction apparatus | Suction apparatus (mucus abstractor) available, observed, and functioning in the delivery area. |
| Manual vacuum extractor | Manual vacuum extractor available, observed, and functioning in the delivery area. |
| Vacuum aspirator or Dilation and Curettage kit | Vacuum aspirator or D&C kit available, observed, and functioning, in the delivery area |
| Partograph | Partograph available, observed, and functioning in delivery area. |
| Disposable latex gloves | Disposable latex gloves observed in delivery area |
| Newborn bag and mask | Newborn bag and mask (AMBU bag and mask) available, observed, and functioning in the delivery area |
| Infant scale | Infant scale observed and functioning in delivery area. |
| Blood pressure apparatus (manual/digital) | Manual or digital blood pressure apparatus observed and functioning in delivery area |
| Handwashing soap and running water or hand disinfectant | Hand-washing soap and running water or hand disinfectant available and observed in delivery area. |
| **Domain E: Medicines and Commodities** | |
| Injectable antibiotic | Injectable antibiotics observed in delivery area and at least one dose valid. |
| Hydrocortisone available at the facility | Hydrocortisone observed at the facility and at least one dose valid. |
| Injectable uterotonic | Oxytocin observed in delivery area with at least one dose valid |
| Skin disinfectant | Skin disinfectant available for newborns in delivery area |
| Magnesium sulphate | Magnesium sulphate available in delivery area with at least one dose valid |
| IV solution with infusion set | IV solution with infusion set available in delivery area with at least one set valid |
| Chlorhexidine for cord cleaning | Chlorhexidine solution (4%) for umbilical cord cleaning available in delivery area, with at least one dose valid. |
| Antibiotic eye ointment for newborn | Tetracycline eye ointment for newborn available in delivery area and at least one dose valid |
| **Domain F: Guidelines, staff training and supervision** | |
| Guidelines: Integrated Management of Pregnancy and Childbirth (IMPAC) Guidelines | Guidelines available in delivery area |
| Comprehensive EmOC Guidelines | Guidelines available in delivery area |
| Guidelines: Guidelines for management of pre-term labor | Guidelines available in delivery area |
| Guidelines for standard precautions | Guidelines available in delivery area |
| Training in neonatal resuscitation | At least one provider of delivery/newborn care in facility received training in the past 24 months |
| Training in early and exclusive breastfeeding | Same as above |
| Training in newborn infection management (including injectable antibiotics) | Same as above |
| Training in thermal care | Same as above |
| Training in cord care | Same as above |
| Training in IMPAC | Same as above |
| Training in routine care during labor and delivery | Same as above |
| Training in Comprehensive EmOC | Same as above |
| Training in Active Management of Third Stage of Labor (AMTSL) | Same as above |
| Training in Kangaroo Mother Care (KMC) | Same as above |
| Supervision | At least half of interviewed providers reported being personally supervised at least once during the 6 months preceding the survey |

**Source:** Wang W, Mallick L, Allen C, Pullum T. Effective coverage of facility delivery in Bangladesh, Haiti, Malawi, Nepal, Senegal, and Tanzania. PloS one. 2019 Jun 11;14(6):e0217853.

*****The measures were obtained from both the facility and provider datasets
